# Diabetes care providers’ opinions and working methods after four years of experience with a diabetes patient web portal; a survey among health care providers in general practices and an outpatient clinic

**DOI:** 10.1186/s12875-018-0781-y

**Published:** 2018-06-21

**Authors:** Maaike C. M. Ronda, Lioe-Ting Dijkhorst-Oei, Rimke C. Vos, Guy E. H. M. Rutten

**Affiliations:** 1 Julius Centre for Health Sciences and Primary Care, University Medical Centre Utrecht, STR 6.131, PO Box 85500, 3508 Utrecht, GA Netherlands; 2 Department of Internal Medicine, Meander Medical Centre, Maatweg 3, 3813 Amersfoort, TZ Netherlands

**Keywords:** Patient web portal, E-health, Communication, Physician attitudes, Diabetes portal, Diabetes self-management

## Abstract

**Background:**

To gain insight into the opinions and working methods of diabetes care providers after using a diabetes web portal for 4 years in order to understand the role of the provider in patients’ web portal use.

**Methods:**

Survey among physicians and nurses from general practices and an outpatient clinic, correlated with data from the common web portal.

**Results:**

One hundred twenty-eight questionnaires were analysed (response rate 56.6%). Responders’ mean age was 46.2 ± 9.8 years and 43.8% were physicians. The majority was of opinion that the portal improves patients’ diabetes knowledge (90.6%) and quality of care (72.7%). Although uploading glucose diary (93.6%) and patient access to laboratory and clinical notes (91.2 and 71.0%) were considered important, these features were recommended to patients in only 71.8 and 19.5% respectively. 64.8% declared they informed their patients about the portal and 45.3% handed-out the information leaflet and website address. The portal was especially recommended to type 1 diabetes patients (78.3%); those on insulin (84.3%) and patients aged< 65 years (72.4%). Few found it timesaving (21.9%). Diabetes care providers’ opinions were not associated with patients’ portal use.

**Conclusions:**

Providers are positive about patients web portals but still not recommend or encourage the use to all patients. There seems room for improvement in their working methods.

**Electronic supplementary material:**

The online version of this article (10.1186/s12875-018-0781-y) contains supplementary material, which is available to authorized users.

## Background

The burden of diabetes is rapidly increasing worldwide [1]. Patient web portals are of interest in this respect and many studies focused on the use of portals by patients with diabetes [2, 3]. A patient portal is a secure online website that gives a person access to his or her personal medical information derived from the physician’s electronic medical record. Portals have shown a range of benefits, such as improved diabetes outcomes, increased patient satisfaction and patient-provider communication, and reduced office visits [4–8]. However, the number of patients that use a portal is low [9–13].

We demonstrated that patients’ unawareness of the existence of a portal is an important barrier for starting its use [14]. So the role of the diabetes care provider seems of importance in the use of patient portals. However, healthcare providers are often also unaware of the existence of a patient portal or of its features [15, 16]. They may underestimate the number of patients that are actually interested in using it [15], are hesitant to start a web-based communication [17], or expect problems with the communication or in the relationship with their patients [18–20]. There is fear for patients experiencing problems with the interpretation of a portal’s data [18, 21, 22], pessimism about patients’ motivation and ability to maintain a personal health record [16], and fear for an increase in the physician’s workload [23–25]. Concerns about reliability, confidentiality, and security of information are other commonly mentioned barriers [20, 24, 26–28]. However, information about the interaction with patients with regard to portal use is lacking and more insight into the daily practice role of the diabetes care provider in this respect seems warranted.

We aimed to gain insight into the opinions and working methods of diabetes care providers after having used for 4 years a diabetes specific electronically medical record in which patients have full access (“web portal”). The following research questions are addressed:

1. What are the opinions of the diabetes care providers about the portal and its functionality?
2. How do they communicate the possibilities of the portal and to which patients?
3. What are the perceived consequences of the portal?
4. Are provider characteristics and opinions associated with the patients’ portal use?

## Methods

### Study setting

“Diamuraal” is a so-called care group, that coordinates the care of patients with diabetes [29, 30]. Within this care group there are 62 primary care practices working (with general practitioners and nurse practitioners) and one outpatient clinic (with internists with subspecialty diabetology or nephrology and specialized diabetes nurses). All practices and providers use the same type of diabetes electronic medical record (EMR). The diabetes EMR is used simultaneously with and besides the general EMR of both the primary and secondary care practices.

### The patient web portal

The general EMR has no portal option, but patients can request a login to access their personal diabetes EMR, via a web portal that provides access to information about the consultation, laboratory results, the so-called ‘problem list’, treatment goals, as well as to general diabetes information and to an overview of all individual diabetes related examinations and consultations that are needed and/or scheduled. Patients can upload glucose levels measured at home, including comments, and are asked for explanations in case of high and low glucose values (“glucose diary”). They can also contact their physician or nurse by secured electronic messaging. In addition, quarterly monitoring office visits can be substituted by self-monitoring; in that case, the diabetes care provider schedules for a patient to complete a standardized check list in his diabetes EMR. The portal is supplementary; all patients receive regular diabetes care according to the Dutch guidelines [31].

### Study design and measures

A postal questionnaire was sent to all 228 diabetes care providers working in Diamuraal. It contained questions about their opinions about the portal and its functionality, to which patients they recommend or discourage the portal’s use, how they communicate the possibilities of the portal with the patients and how they perceive the consequences of the portal, not only with regard to patient self-management but also for the healthcare provider. Twenty-six questions had to be scored on a 5-point Likert scale, 15 questions on a 3-point Likert scale, eight questions were multiple choice and one was open ended. In addition, six items about provider characteristics were included (see Additional file 1 for an overview). All issues addressed in the questions were proven relevant based on literature [2, 32] and it was pilot tested by 2 general practitioners, an internist and two diabetes nurses from the Diamuraal care group.

Possible respondents received a reminder twice in a 3 week interval; the first by post, the second by telephone. From the central database of Diamuraal, data were collected about the number of patients with access to the patient portal per practice and about the start date of practices joining Diamuraal.

### Statistical analysis

Categorical variables were expressed as counts with percentages and continuous variables as means with standard deviation (SD). Continuous variables were checked for normality. The characteristics and opinions of different type of health care providers were compared with chi-square tests for categorical and unpaired t-tests for continuous variables. Items with a 5-point Likert scale were transformed into three answer categories by combining the two highest and the two lowest response categories. Linear regression was used to assess the association between the number of patients with a login request and the time the practice had been using the portal, and Spearman’s rho was used to assess the correlation between provider’s opinions and the number of patients with a login request per practice. Data were analysed using SPSS for Windows (version 21, SPSS Inc., Chicago, IL, USA).

## Results

In total 129 (56.6%) diabetes care providers completed the questionnaire. One questionnaire was excluded because of > 10% missing values, so 128 questionnaires were analysed. Responders were more often female (75% of participants vs 49% of non-responders, *p* < 0.001) and had a higher proportion of patients with access to the portal in their practices, although the difference was not significant (17.6 ± 11.4% versus 7.9 ± 6.4% (*p* = 0.07).

Respondents’ mean age was 46.2 ± 9.8 years (Table 1). On average 157.8 ± 9.1 diabetes patients were treated in a primary care practice (range 52–508); the outpatient clinic treated 2647 diabetes patients. The outpatient clinic had a higher percentage of patients with access to the portal than the primary care practices (52.8% versus 16.9%). The diabetes EMR with portal was used for 5 years by the outpatient clinic compared to on average 3.8 years in primary care. The medical specialties invited had a differential response rate, ranging from 100% (internists and diabetes nurses) to 76.8% (nurse practitioners) and 35.7% (GPs).

**Table 1:** Characteristics of Responders (*N* = 128)

	General Practitioner	Nurse Practitioner	Internist	Diabetes Nurse
Number	45	56	11	16
Gender, male	27 (60,0%)	0 (0%)	6 (54,5%)	0 (0%)
Age, years ± SD	51.4 ± 12.8	43.2 ± 9.9	46.4 ± 10.8	49.5 ± 10.3

### Opinions about the portal and its functionality

The two main reasons for respondents to work with the portal was because they felt that it could improve the quality of diabetes care (77/128 providers, 60.2%) and the supposed improvement of communication between the different members of the diabetes team by working with one common medical record (56/128, 43.8%). Most respondents were positive about the use of a patient portal with respect to the quality of care, patient self-care and consult preparation. However, although most respondents (strongly) agreed that the provided diabetes information on the portal could lead to improved self-management, only 20% thought that it would improve self-management in three quarters of their own patients. In general the internists were more sceptical about the portal, but differences between type of health care provider were not significant (Table 2). Most respondents scored the glucose diary (117/125, 93.6%) and the access to laboratory values and treatment goals for the patients (114/125, 91.2%) as (very) important features of the portal. Other features that were scored as (very) important were the possibility to send an e-message (98/124, 79.0%) and the patient’s access to clinical notes (88/124, 71.0%). About two out of three (66.9%) respondents scored web-based scheduling consultations (very) important, the same applied to ‘insight in the personal care team’ (67.7%) and diabetes information (64.8%). Insight into prescribed medication was scored as (very) important by 61.8% of the respondents. Suggestions for improvement of the portal mainly regarded the glucose diary (“difficult to fill in for patients with insulin-pump therapy”), the option to add self-measured blood pressure levels by the patient (which actually was an existing feature, but apparently not known by most diabetes care providers working with this portal), adding of other non-diabetes related laboratory values or patient characteristics (e.g. history, type of work and current diet), and tailored diabetes and medication information.

**Table 2:** Opinions about the possible effects of the diabetes web portal. Percentages of respondents

“I (strongly) agree that…..”	All providers	General practitioner	Nurse practitioner	Internist	Diabetes nurse	
	(*N* = 128)	(*n* = 45)	(*n* = 56)	(*n* = 11)	(*n* = 16)	*P*-value
a patient portal improves the quality of diabetes care	72.7	77.8	67.9	63.6	81.3	0.60
a patient portal can prevent medical mistakes	55.5	60.0	50.0	45.5	68.8	0.33
the diabetes knowledge that patients gain through the portal can lead to improved self-management	90.6	97.7	85.5^a^	81.8	100	0.30
a positive effect of the patient web portal is that patients can prepare themselves to the diabetes consultation	71.1	73.3	67.9	63.6	81.3	0.76
the use of a patient portal can lead to better self-management in three quarters of my patients	20.3	24.4	16.1	9.1	31.3	0.11
in a cardiometabolically well-controlled patient with portal access, one of the quarterly monitoring visits can be substituted by self-monitoring	69.5	68.9	73.2	45.5	75.0	0.10

^a^1 answer missing

### How do diabetes care providers communicate the portal, and to which patients?

**Fig. 1:**
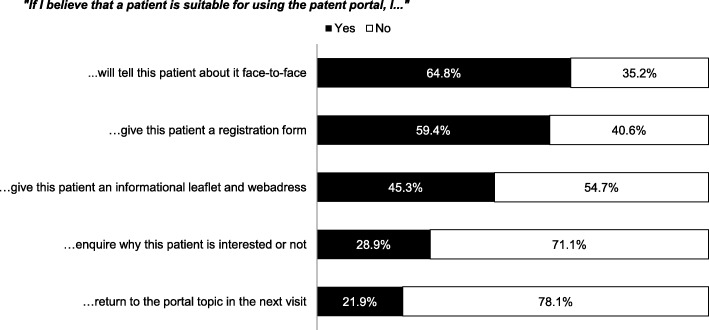
How to discuss the option of using the portal with patients

Most often the face-to-face method was reported as to communicate the use of the portal. Additional types of informing the patient and communication about the portal were less often utilized (Fig. 1). More than half of the respondents reported that they always or regularly encourage their patients to use the portal for adding glucose values as well as for e-messaging. Preparing a consultation and re-reading the information before and after a consultation were least encouraged (Table 3). Respondents answered that they recommend the portal to most of their patients, but especially to patients with type 1 diabetes mellitus, patients on insulin therapy, younger and higher educated patients (Table 4). Diabetes care providers did not differ in this respect (data not shown).

**Table 3:** Encouragement to patients to use certain portal features. Percentages of respondents

“To which extent do you encourage your patient to…”	N	Always or regularly	Sometimes	Rarely or never
send you an electronic message through the portal	122	55.7	15.6	28.7
upload glucose values more often	124	71.8	16.9	11.3
re-read information after a consultation	123	30.1	32.5	37.4
prepare for a consultation by viewing laboratory results and agreed targets	123	19.5	43.1	37.4
inform you when he/she experiences a problem with the portal	123	43.9	26.0	30.1
tell you when the meaning of laboratory values is unclear	123	36.6	27.6	35.8
tell you when medical phrasings used in the health record are unclear	123	39.0	27.6	33.3
turn to you if he/she has questions about self-management	124	47.6	26.6	25.8

**Table 4:** To what patients do the providers recommend the diabetes portal? Percentages of respondents

	N	Recommend	Neutral	Discourage
Patients with type 1 diabetes mellitus	115	78.3	20.9	0.9
Patients with type 2 diabetes mellitus	125	60.8	39.2	0.0
Patients with good cardiometabolic control	126	65.9	34.1	0.0
Patients with poor cardiometabolic control	126	63.5	27.9	8.6
Patients who do not use diabetes-specific medication	126	28.6	63.5	7.9
Patients who use oral diabetes medication	127	51.2	45.7	3.1
Patients who use insulin	127	84.3	15.7	0.0
Patients without comorbidities	127	55.1	43.3	1.6
Patients with comorbidities	127	52.8	40.2	7.1
Patients without language barriers	126	69.0	30.5	0.0
Patients with language barriers	125	10.2	39.8	47.7
Patients with lower education	126	25.0	48.4	25.0
Patients with higher education	125	73.6	26.4	0.0
Patients < 65 years	127	72.4	27.6	0.0
Patients > 65 years	125	33.6	54.7	9.4

### Perceived consequences for the care provider

One third of the diabetes care providers (40/121, 33.1%) declared that the provider’s role in the treatment (strongly) improved, whereas two-thirds of the providers (82/121, 67.8%) felt that the involvement of the patient in the treatment (strongly) improved. Other perceived (strong) improvements were the collaboration with the patient (85/121, 70.2%) and the increased knowledge of patients about diabetes mellitus (70/120, 58.3%). The majority of respondents stated that having access to the EMR stimulates self-management and self-correcting behaviour of patients. Most reported that they did not change their way of medical notation and most also stated that the frequency of patient’s personal consultations had not changed after the introduction of the portal (Table 5).

**Table 5:** Perceived consequences of working with the diabetes patient portal. Percentages of respondents

	N	Yes (%)
“Access to his/her diabetes EMR via a web portal …”	128	
stimulates the self-management and self-correcting behaviour of the patient		75.8
improves communication during consultation with a well prepared patient		44.5
results in saving time		21.9
results in deceased workload		10.9
“I write the medical information...”	128	
as I always did		63.3
in an easier language than before		37.5
with less information than before		7.8
“I think that patients who use the patient portal…”	122	
have an increased frequency of visits		2.5
have an unchanged frequency of visits		80.3
have a decreased frequency of visits		17.2
“How do you feel about patients sending you an e-message?”	128	
(very) positive		65.6
neutral		28.1
(very) negative		6.3
“How many e-messages do you receive per week?”	124	
0 messages		33.1
1–10 messages		59.7
≥ 11messages		7.3
“Who usually answers the e-message of patients?”	80^a^	
the health care provider answers only the messages of his/her own patients		31.3
the physician (GP or internist) answers all messages		2.5
the nurse (nurse practitioner or diabetes nurse) answers all messages		66.3

^a^All respondents who receive e-messages

### Are provider characteristics and opinions associated with the patients’ portal use?

The proportion of patients with access to the portal was not related to the number of years the practice had been using the portal (beta 0.32 (95% CI -0.15 – 0.78), *p* = 0.17). Except for the statement that it can lead to improved self-management in general (r_s_-.296, *p* = 0.03), the respondents’ opinion about each of the six possible effects of the portal as mentioned in Table 2 was not associated with the proportion of patients within the practice that requested a login to the portal (improving the quality of diabetes care r_s_ − .009, *p* = 0.95; preventing medical mistakes r_s_.003, *p* = 0.99; patients being more prepared during consultation r_s_ − .164, *p* = 0.22; improving self-management in own patients r_s_ − .211, *p* = 0.12; substitute a quarterly control by self-control r_s_-.174, *p* = 0.20).

## Discussion

The current study explored the opinions of diabetes care providers on the usefulness of an existing web portal and their working methods with regard to the web portal. They feel it could improve the quality of diabetes care and self-management of patients, but do not recommend it to all of their patients. They mostly explain the use of the portal directly with the patient, but they do not provide additional written information nor inquire into the patient’s view. The level of active encouragement of specific portal features is low, even when physicians or nurses feel those features are important. Both nurses and physicians are selective in promoting the portal. The suggestion that web portals may save time for the diabetes care provider seems not justified.

Several previous surveys have indicated that health care providers are reluctant to encourage patients to gain access to all medical notes; sometimes they considered patient health records more as a resource for physicians than a tool for patients [16, 33]. Physicians expected that patients’ access to physician notes would result in greater worry among patients and that they anticipated more questions by patients [19], while afterwards these expectations did not become reality [21], and patients felt that access to physician notes led to an improved understanding, a better relationship with their provider and improved quality of care and self-care [34]. Such a gap between physicians’ expectations on how patients will perceive the use of a web portal and the actual patients ‘experience might hinder providers’ enthusiasm of discussing a portal with all their patients. Furthermore, health care providers may have insufficient knowledge on the best ways to make use of a web portal as an addition to current diabetes care and they may lack the necessary skills to stimulate patients.

In contrast to what many patients stated about their unawareness of this diabetes portal [14], the majority of the diabetes care providers reported that they informed their patients about the portal, most often face-to face. However, they rarely address it during the next visit, which might have caused the discrepancy between patients’ and providers’ answers. It is known that general practitioners rarely assess their diabetic patients’ recall or comprehension of new concepts [35]. From the current study we cannot explain why diabetes care providers appreciate for example the portal’s glucose diary and patients preparing a consult with the use of the portal, but only encourage the use of these features on a limited scale. With the glucose diary can patients not only upload their glucose levels measured at home, but also must add information to clarify why levels are too high or too low. This is valuable information for the physician who can give the patient subsequent feedback and can also contribute to more self-awareness in patients. Additional training might be necessary to support the providers in discussing the benefits of this with patients, including helping with and checking the patients understanding of the information. Also lack of time might be a reason for the working methods of the diabetes care providers. They perceived no benefits of the portal in terms of time saving and a decreased workload. Patients’ office visit frequency was estimated to have remained similar by most respondents, and this perception is likely to be correct. Other studies led to an increase of both e-messaging and telephone encounters between patients and provider [36, 37], whereas in a study in the USA, patients actually turned to their portals after visits, and portal use did not lead to an increase in primary care visits [38]. We expect that with more experience with the full range of possibilities a patient web portal has to offer, the workload may ultimately decrease as patients will start to use the portal for substitution of care.

Despite the positive attitude of our respondents towards the portal for patient use, only 17.6% of their patients had requested access to the portal. We did not find an association between the opinion of a healthcare provider and the proportion of patients within the practice that requested a login to the portal. These findings suggest that other factors determine whether patients will use the diabetes portal, e.g. insulin use, hypoglycaemic episodes and diabetes knowledge. We did find that diabetes nurses are most optimistic about the portal, while the medical specialists at the same hospital are more sceptical. They both treat the same complex patients who are more likely to request a login [39]. This difference of opinions between type of providers within the same setting might be a reason we did not find an association between positive opinions and proportion of patients within the practice that requested a login. Furthermore, it is also possible that health care providers were more positive about the portal in our questionnaire while in daily use they hold a different opinion and therefore do not recommend it more often. Another possibility is that they are positive but due to e.g. time constraints during consultation do not recommend portal use more actively. We might need to stimulate the providers to play a more active role to increase the number of patients with a login to the portal.

### Strengths and limitations of this study

The strength of our study is that we evaluated a web portal that has been in use in daily practice for 4 years. However, several limitations should be considered. First, we have a relatively small surveyed population. Response rates of physician surveys are notoriously low and our rate is comparable to others [40]. One of the researchers works as an internist at the hospital. She had no access to the returned questionnaires, but her position might have influenced the response rate among the diabetes nurses. However, we have no reason to assume that this position influenced the outcomes of the survey. Second, significantly fewer general practitioners responded. However, we found no difference of opinions between general practitioners and internists. Third, the tendency that respondents had a higher percentage of patients with access to the portal than non-respondents might indicate a selection-bias. It is possible that general practitioners who did not return the questionnaire are less positive about using a patient web portal. Finally, our questionnaire was designed based of determinants of patient portal use from literature. It was evaluated by experts but we might have missed information which could have been found if alternative methodologies, such as in-depth interviews, were used. For example, the discrepancy between health care providers’ opinions about the portal leading to improvement of self-management and the low number of providers expecting that three quarters of their own patients were able to use the portal to improve their diabetes self-management, might have been the result of the wording (‘three quarters’) in the questionnaire. It would have been better to phrase it as a more open question.

### Implications for clinical practice and further research

Despite positive opinions about the possible effects of a diabetes web portal, diabetes care providers do not offer maximal support and encouragement to patients that are likely necessary to increase the portal use and its possible benefits. They merely discuss the portal with patients face-to-face, hardly provide additional information and hardly check if patients understand how they could benefit from portal use. May be if providers will receive additional training in this respect, the gap between their opinions and their working methods can become smaller. Such training can include teaching care providers how to explore patients’ motivation and how to support patients in maintaining their health record and interpreting their data, as well as addressing anticipated problems in electronic communication and the provider-patient relationship. Furthermore, as a result of this study, we are considering adjustments to this web portal to tailor the portal for different categories of patients, for example for patients who use insulin and those who do not.

## Additional file

Additional file 1: Additional information accompanies this article (DOCX 32 kb).

## References

[1] WHO (2014). Global status report on noncommunicable diseases.

[2] Andreassen HK, Bujnowska-Fedak MM, Chronaki CE, Dumitru RC, Pudule I, Santana S, Voss H, Wynn R (2007). European citizens’ use of E-health services: a study of seven countries. BMC Public Health.

[3] Osborn CY, Mayberry LS, Mulvaney SA, Hess R (2010). Patient web portals to improve diabetes outcomes: a systematic review. Curr Diab Rep.

[4] Zhou YYG, Garrido T, Chin HL, Wiesenthal AM, Liang LL (2007). Patient access to an electronic health record with secure messaging: impact on primary care utilization. Am J Manag Care.

[5] Ralston JD, Hirsch IB, Hoath J, Mullen M, Cheadle A, Goldberg HI (2009). Web-based collaborative care for type 2 diabetes: a pilot randomized trial. Diabetes Care.

[6] Holbrook A, Thabane L, Keshavjee K, Dolovich L, Bernstein B, Chan D, Troyan S, Foster G, Gerstein H (2009). Individualized electronic decision support and reminders to improve diabetes care in the community: COMPET5rfcE II randomized trial. CMAJ.

[7] McCarrier KP, Ralston JD, Hirsch IB, Leweis G, Martin DP, Zimmerman FJ, Goldberg HI (2009). Web-based collaborative care for type 1 diabetes**:** a pilot randomized trial. Diabetes Technol Ther.

[8] Wald JS, Businger A, Gandhi TK, Grant RW, Poon EG, Schnipper JL, Volk LA, Middleton B (2010). Implementing practice-linked pre-visit electronic journals in primary care: patient and physician use and satisfaction. J Am Med Inform Assoc.

[9] Greenhalgh T, Hinder S, Stramer K, Bratan T, Russell J (2010). Adoption, non-adoption, and abandonment of a personal electronic health record: case study of HealthSpace. BMJ.

[10] Weppner WG, Ralston JD, Koepsell TD, Grothaus LC, Reid RJ, Jordan L, Larson EB (2010). Use of a shared medical record with secure messaging by older patients with diabetes. Diabetes Care.

[11] Sarkar U, Karter AJ, Liu JY, Adler NE, Nguyen R, Lopez A, Schillinger D (2011). Social disparities in internet patient portal use in diabetes: evidence that the digital divide extends beyond access. J Am Med Inform Assoc.

[12] Yamin CK, Emani S, Williams DH, Lipsitz SR, Karson AS, Wald JS, Bates DW (2011). The digital divide in adoption and use of a personal health record. Arch Intern Med.

[13] Black H, Gonzalez R, Priolo C, Schapira MM, Sonnad SS, Hanson CW, Langlotz CP, Howell JT, Apter AJ (2015). True “meaningful use”: technology meets both patient and provider needs. Am J Manag Care.

[14] Ronda MC, Dijkhorst-Oei LT, Rutten GE (2014). Reasons and barriers for using a patient portal: survey among patients with diabetes mellitus. J Med Internet Res.

[15] Fuji KT, Galt KA, Serocca AB (2008). Personal health record use by patients as perceived by ambulatory care physicians in Nebraska and South Dakota: a cross-sectional study. Perspect Health Inf Manag.

[16] Witry MJ, Doucette WR, Daly JM, Levy BT, Chrischilles EA (2010). Family physician perceptions of personal health records. Perspect Health Inf Manag.

[17] Hassol A, Walker JM, Kidder D, Rokita K, Young D, Pierdon S, Deitz D, Kuck S, Ortiz E (2004). Patient experiences and attitudes about access to a patient electronic health care record and linked web messaging. J Am Med Inform Assoc.

[18] Zickmund SL, Hess R, Bryce CL, McTigue K, Olshansky E, Fitzgerald K, Fischer GS (2008). Interest in the use of computerized patient portals: role of the provider-patient relationship. J Gen Intern Med.

[19] Walker J, Leveille SG, Ngo L, Vodicka E, Darer JD, Dhanireddy S, Elmore JG, Feldman HJ, Lichtenfeld MJ, Oster N (2011). Inviting patients to read their doctors’ notes: patients and doctors look ahead: patient and physician surveys. Ann Intern Med.

[20] Crotty BH, Mostaghimi A, Landon BE (2013). Preparing residents for future practice: report of a curriculum for electronic patient-doctor communication. Postgrad Med J.

[21] Delbanco T, Walker J, Bell SK, Darer JD, Elmore JG, Farag N, Feldman HJ, Mejilla R, Ngo L, Ralston JD (2012). Inviting patients to read their doctors’ notes: a quasi-experimental study and a look ahead. Ann Intern Med.

[22] Callen J, Giardina TD, Singh H, Li L, Paoloni R, Georgiou A, Runciman WB, Westbrook JI (2015). Emergency physicians’ views of direct notification of laboratory and radiology results to patients using the internet: a multisite survey. J Med Internet Res.

[23] Miller DP, Latulipe C, Melius KA, Quandt SA, Arcury TA (2016). Primary care Providers’ views of patient portals: interview study of perceived benefits and consequences. J Med Internet Res.

[24] Kittler AF, Carlson GL, Harris C, Lippincott M, Pizziferri L, Volk LA, Jagannath Y, Wald JS, Bates DW (2004). Primary care physician attitudes towards using a secure web-based portal designed to facilitate electronic communication with patients. Inform Prim Care.

[25] Keplinger LE, Koopman JK, Mehr DR, Kruse RL, Wakefield DS, Wakefield BJ, Canfield SM (2013). Patient portal implementation: resident and attending physician attitudes. Fam Med.

[26] Zwaanswijk M, Verheij RA, Wiesman FJ, Friele RD (2011). Benefits and problems of electronic information exchange as perceived by health care professionals: an interview study. BMC Health Serv Res.

[27] Bell SK, Mejilla R, Anselmo M, Darer JD, Elmore JG, Leveille S, Ngo L, Ralston JD, Delbanco T, Walker J (2016). When doctors share visit notes with patients: a study of patient and doctor perceptions of documentation errors, safety opportunities and the patient-doctor relationship. BMJ Qual Saf.

[28] Kruse CS, Argueta DA, Lopez L, Nair A (2015). Patient and provider attitudes toward the use of patient portals for the management of chronic disease: a systematic review. J Med Internet Res.

[29] Campmans-Kuijpers MJ, Baan CA, Lemmens LC, Rutten GE (2015). Change in quality management in diabetes care groups and outpatient clinics after feedback and tailored support. Diabetes Care.

[30] Struijs JN, Baan CA (2011). Integrating care through bundled payments - lessons from the Netherlands. N Engl J Med.

[31] Rutten GEHM, De Grauw WJ, Nijpels G, Houweling ST, Van de Laar FA, Bilo H, Holleman F, Burgers JS, Wiersma TJ, PGH J (2013). NHG-Standaard Diabetes Mellitus type 2 (derde herziening). Huisarts Wet.

[32] Weingart SN, Rind D, Tofias Z, Sands DZ (2006). Who uses the patient internet portal? The PatientSite experience. J Am Med Inform Assoc.

[33] Grunloh C, Cajander A, Myreteg G (2016). “The record is our work tool!”-Physicians’ framing of a patient portal in Sweden. J Med Internet Res.

[34] Esch T, Mejilla R, Anselmo M, Podtschaske B, Delbanco T, Walker J (2016). Engaging patients through open notes: an evaluation using mixed methods. BMJ Open.

[35] Schillinger D, Piette J, Grumbach K, Wang F, Wilson C, Daher C, Leong-Grotz K, Castro C, Bindman AB (2003). Closing the loop: physician communication with diabetic patients who have low health literacy. Arch Intern Med.

[36] Liss DT, Reid RJ, Grembowski D, Rutter CM, Ross TR, Fishman PA (2014). Changes in office visit use associated with electronic messaging and telephone encounters among patients with diabetes in the PCMH. Ann Fam Med.

[37] Dexter EN, Fields S, Rdesinski RE, Sachdeva B, Yamashita D, Marino M (2016). Patient-provider communication: does electronic messaging reduce incoming telephone calls?. J Am Board Fam Med.

[38] Leveille SG, Mejilla R, Ngo L, Fossa A, Elmore JG, Darer J, Ralston JD, Delbanco T, Walker J (2016). Do patients who access clinical information on patient internet portals have more primary care visits?. Med Care.

[39] Ronda MC, Dijkhorst-Oei LT, Rutten GE (2015). Patients’ experiences with and attitudes towards a diabetes patient web portal. PLoS One.

[40] Cook JV, Dickinson HO, Eccles MP (2009). Response rates in postal surveys of healthcare professionals between 1996 and 2005: an observational study. BMC Health Serv Res.

