# Supplementary material for: Diabetes care providers’ opinions and working methods after four years of experience with a diabetes patient web portal; a survey among health care providers in general practices and an outpatient clinic
Source: BMC Fam Pract. 2018 Jun 21;19:94. doi: 10.1186/s12875-018-0781-y (PMC6013979; doi:10.1186/s12875-018-0781-y)
Supplement: Supplementary file 1 — Additional information accompanies this article (DOCX 32 kb). [file 12875_2018_781_MOESM1_ESM.docx]

**Questionnaire**

**01. Why have you chosen for working with an EMR with patient portal?** *(more than one option*

*possible)*

□ Decrease workload by substituting one of the quarterly visits at the clinic in a self-control by the

patient.

□ Optimizing coordination with co-workers due to working together in one record.

□ Improvement of quality of diabetes care.

□ A majority of my co-workers within our practice wanted to work with this system.

□ To operate efficiently I chose to connect with the care group and I had to accept the type of

system.

□ Another reason.

**02. The following statements are about the electronic medical record (EMR) and about self-management. You can give one answer per row, according to your preferences.**

|  | Strongly agree | Agree | Neither agree or disagree | Disagree | Strongly disagree |
| --- | --- | --- | --- | --- | --- |
| A patient portal improves the quality of diabetes care | □ | □ | □ | □ | □ |
| A patient portal can prevent medical mistakes | □ | □ | □ | □ | □ |
| The diabetes knowledge that patients gain through the portal can lead to improved self-management | □ | □ | □ | □ | □ |
| A positive effect of the patient web portal is that patients can come prepared to the consultation (e.g. by taking a print-out of their own medical record) | □ | □ | □ | □ | □ |
| The use of a patient portal can lead to better self-management in three quarters of my patients | □ | □ | □ | □ | □ |
| In a cardiometabolically well-controlled patient with portal access, one of the quarterly controls can be substituted by a self-control | □ | □ | □ | □ | □ |

**03. What part of the patient web portal do you believe is important (for the patient)?**

The patient….

|  | Very important | Important | Neutral | Not important | Not at all important |
| --- | --- | --- | --- | --- | --- |
| … can see which controls are still needed | □ | □ | □ | □ | □ |
| … has an overview of all health care providers involved in the treatment | □ | □ | □ | □ | □ |
| … can reread the information provided during consultation | □ | □ | □ | □ | □ |
| … has access to his/her data (e.g. laboratory) | □ | □ | □ | □ | □ |
| … can upload a glucose diary | □ | □ | □ | □ | □ |
| … can send me a secured e-message | □ | □ | □ | □ | □ |
| … has an summary of his/her medications | □ | □ | □ | □ | □ |
| … can use the portal for general diabetes information | □ | □ | □ | □ | □ |

**04. Are there features that would improve the patient portal?**

….. *(open text)*

**05. If I believe a patient is suitable for working with a patient portal, than I…** *(more than one option*

*possible)*

□ will tell the patient

□ give this patient an informational leaflet and web-address

□ return to this in a next visit

□ enquire why this patient is interested or not

□ give this patient the registration form

**06. To which extend do you encourage your patients to use the following patent portal features:**

|  | always | often | sometimes | rarely | never |
| --- | --- | --- | --- | --- | --- |
| Send you an electronic message through the portal | □ | □ | □ | □ | □ |
| Upload a glucose-diary more often | □ | □ | □ | □ | □ |
| Reread information after consultation | □ | □ | □ | □ | □ |
| Prepare for a consult by viewing laboratory results and agreed targets | □ | □ | □ | □ | □ |
| To inform you when he/she experience a problem with the portal | □ | □ | □ | □ | □ |
| Tell you when the meaning of laboratory values is unclear | □ | □ | □ | □ | □ |
| Tell you when medical phrasings used are unclear | □ | □ | □ | □ | □ |
| Turn to you if he/she has questions about self-management | □ | □ | □ | □ | □ |

**07. Do you recommend or discourage the portal in the patients mentioned below?**

|  | recommend | Neutral | discourage |
| --- | --- | --- | --- |
| Patients with type 1 diabetes mellitus | □ | □ | □ |
| Patients with type 2 diabetes mellitus | □ | □ | □ |
| Patients with good cardiometabolic control | □ | □ | □ |
| Patients with poor cardiometabolic control | □ | □ | □ |
| Patients who do not use diabetes-specific medication | □ | □ | □ |
| Patients who use oral diabetes medication | □ | □ | □ |
| Patients who use insulin | □ | □ | □ |
| Patients with no comorbid conditions | □ | □ | □ |
| Patients with comorbid conditions | □ | □ | □ |
| Patients without language barriers | □ | □ | □ |
| Patients with language barriers | □ | □ | □ |
| Patients with a lower education | □ | □ | □ |
| Patients with a higher education | □ | □ | □ |
| Patients who are younger (< 65 years) | □ | □ | □ |
| Patients who are older ( 65 years) | □ | □ | □ |

**08. In regard to the diabetes treatment of your patients who are using the patient portal:**

|  | Strongly improved | Somewhat improved | Not improved, not worse | Somewhat worse | Strongly worse |
| --- | --- | --- | --- | --- | --- |
| Is my own role in the treatment of these patients… | □ | □ | □ | □ | □ |
| Is the own role of the patient in the treatment… | □ | □ | □ | □ | □ |
| Is the collaboration with the patient in regard to treatment… | □ | □ | □ | □ | □ |
| De knowledge about diabetes of patients who use the patient portal is… | □ | □ | □ | □ | □ |

**09. What perceived benefits or problems are there for you in regard to patients having access to his/her EMR?** *(more than one option possible)*

□ It results in decreased workload

□ It results in saving time

□ It stimulates the self-management and self-correcting behaviour of the patient

□ It improves communication during consultation with a well-prepared patient

□ other… *(plain text)*

**10. Through the portal, your patient can reread the information you written during consultation.**

**Does that have consequences for the way you write the information in the EMR or does it have**

**consequences for the amount and type of information you write down?** *(more than one option*

*possible)*

□ I write the information as I always did

□ I write less information than before

□ I write in an easier language than before (e.g. simple phrasings, less abbreviations)

**11. Do you believe that patients who use the patient portal…**

□ have an increased frequency of visits

□ have a decreased frequency of visits

□ have an unchanged frequency of visits

**12. How do you feel about patients sending you an e-message?**

□ very positive

□ positive

□ neutral

□ negative

□ very negative

**13. How many e-messages do you receive per week:**

□ 0 messages

□ 1-10 messages

□ ≥ 11 messages

**14. Within your practice, who usually answers the e-message of patients?**

□ We all answer the message of our own patients.

□ One of the physicians (GP or internist) answers all the messages (despite the main health care

provider of the patient).

□ One of the nurse practitioners / diabetes nurses answers all the messages (despite the main health care provider of the patient).

**15. Are you:**

□ family care physician

□ nurse practitioner

□ internist

□ specialized diabetes nurse

**16. I am**

□ male

□ female

**17. My age is…** *(open text)*

**18. The number of patients with diabetes mellitus that are in my care …** *(open text)*

**19. The number of patients with diabetes mellitus within my practice that use the patient portal**

**are:**

□ less than 50

□ between 50-100

□ over 100

**20. How long do you use the EMR with patient portal?**

□ less than 2 years

□ between 2-5 years

□ from the beginning

***Thank you very much for completing this questionnaire***
